# 

**Published:** 1861-04

**Authors:** 


					﻿i	Piibll*lied	at $3 per A nil mil In Advance.
W*	__ ___________ _ -	____ .	r
1	T Z_. jxr T TV 1
*
*
5	*
>»
"	c
Li	€
{ JOUHXAL.	5
5'''-T —	j
i	'
f	. J. G. WESTMORELAND, M. D.,
“ : Professor of Materia Medic a axd Medical Jcrifprvdesce is the Atlanta Medical College, '*
c	EDITOR AND PROPRIETOR.	I
’	s
S	. i
*	ST
*	• ?
b	    Z
3	Pax et scleiitia, ned veritaw slue timore.	•
? . ‘ 2
w
5
L Vol. VI.	APRIL, 1861.	No. VI11. j
c .	*
5 I .	s
b	•*
i	ATLANTA, GA:	2
a	IXTELT.IGEXCRR PRIX'!',	S
; ' 18 0 1. ?
£_ ______ ____________ _ 1’
Each Number contains SIxty-Foiir Pages.
				

